# A internet como fonte de informação em saúde: resultados de um inquérito domiciliar em municípios brasileiros das regiões Sul e Centro-oeste

**DOI:** 10.1590/0102-311XPT102225

**Published:** 2026-03-02

**Authors:** Tatiana da Silva Sempé, Patricia Romualdo de Jesus, Liziane Maahs Flores, Edi Franciele Ries, Paulo Maximiliano Corrêa, Juliane Fernandes Monks da Silva, Marcia Regina Martins Alvarenga, Rogério Dias Renovato, Ruberval Franco Maciel, Sotero Serrate Mengue, Tatiane da Silva Dal Pizzol

**Affiliations:** 1 Universidade Federal do Rio Grande do Sul, Porto Alegre, Brasil.; 2 Universidade Federal de Santa Maria, Santa Maria, Brasil.; 3 Universidade Federal de Pelotas, Pelotas, Brasil.; 4 Universidade Estadual de Mato Grosso do Sul, Dourados, Brasil.; 5 Universidade Estadual de Mato Grosso do Sul, Campo Grande, Brasil.

**Keywords:** Letramento em Saúde, Acesso à Informação, Uso da Internet, Inquéritos e Questionários, Health Literacy, Access to Information, Internet Use, Surveys and Questionnaires, Alfabetización en Salud, Acceso a la Información, Uso de Internet, Encuestas y Cuestionarios

## Abstract

O amplo acesso a informações online favorece a busca por conteúdos em saúde. Os objetivos foram identificar o perfil de uso da internet e o grau de dificuldade na busca por informações em saúde por adultos, descrevendo os principais conteúdos buscados e meios utilizados, além de avaliar a associação com características sociodemográficas, médicas e com o grau de letramento em saúde. Inquérito domiciliar realizado em cinco municípios das regiões Sul e Centro-oeste do Brasil. O questionário incluiu dados demográficos, socioeconômicos, uso da internet e grau de letramento em saúde. Realizaram-se análises descritivas e regressão de Poisson para estimar razões de prevalência. Foram incluídos 1.181 indivíduos, sobretudo mulheres, entre 18 e 39 anos, 8 anos ou mais de escolaridade, classe econômica elevada, raça branca, boa autoavaliação de saúde e letramento em saúde problemático. Do total, 92,3% tinham acesso à internet; desses, 77,1% a utilizavam para buscar informações em saúde. Os temas mais buscados foram sintomas (89,1%) e medicamentos (84,5%). As ferramentas mais utilizadas foram Google (94,6%) e YouTube (41,7%). A maioria relatou facilidade para usar palavras corretas (68,6%) e encontrar informações (70,2%), mas dificuldade em avaliar a confiabilidade (44,8%) e aplicar as informações em decisões de saúde (25,9%). Na análise ajustada, maior escolaridade, ser jovem e níveis mais elevados de letramento em saúde foram associados à busca por informações em saúde online. O uso da internet foi amplamente relatado, apesar das dificuldades em avaliar a confiabilidade e aplicar as informações, destacando a necessidade de conteúdos em saúde online de qualidade e acessíveis.

## Abstract (en)

Wide access to online information favors the search for health content. The aims of the present study were to identify the profile of internet use and degree of difficulty in the search for health information by adults, describing the main contents sought and means used, and to investigate associations with sociodemographic/medical characteristics and degree of health literacy. A household survey was conducted in five municipalities in the South and Central-West regions of Brazil. The questionnaire included demographic and socioeconomic data, internet use, and degree of health literacy. Descriptive analyses and Poisson regression were performed to estimate prevalence ratios. A total of 1,181 individuals were included. Women, age between 18 and 39 years, eight or more years of schooling, high economic class, White race, good self-rated health, and problematic health literacy predominated in the sample. A total of 92.3% had access to the internet and 77.1% of these individuals used the internet to search for health information. The most searched topics were symptoms (89.1%) and medications (84.5%). The search tools most used were Google (94.6%) and YouTube (41.7%). Most participants reported ease in using correct words (68.6%) and finding information (70.2%), but difficulty in assessing reliability (44.8%) and applying information to health-related decisions (25.9%). In the adjusted analysis, a higher education, younger age, and higher levels of health literacy were associated with searching for health information online. The use of the internet was widely reported, despite difficulties in assessing reliability and applying the information. The findings underscore the need for accessible online health content of adequate quality.

## Abstract (es)

El amplio acceso a información en línea favorece la búsqueda de contenidos de salud. Los objetivos fueron identificar el perfil de uso de internet y el grado de dificultad en la búsqueda de información de salud entre adultos, describiendo los principales contenidos buscados y los medios utilizados, así como evaluar la asociación con características sociodemográficas, médicas y con el grado de alfabetización en salud. Encuesta domiciliaria realizada en cinco municipios de las regiones Sur y Centro-oeste de Brasil. El cuestionario incluyó datos demográficos y socioeconómicos, uso de internet y nivel de alfabetización en salud. Se realizaron análisis descriptivos y regresión de Poisson para estimar las razones de prevalencia. Se incluyeron en el estudio 1.181 individuos, en su mayoría mujeres, de edades comprendidas entre 18 y 39 años, con 8 o más años de escolaridad, de clase socioeconómica alta, raza blanca, con buena autopercepción de salud y alfabetización en salud problemática. Del total, el 92,3% tenía acceso a internet; de ellos, el 77,1% lo utilizaba para buscar información de salud. Los temas más buscados fueron síntomas (89,1%) y medicamentos (84,5%). Las herramientas más utilizadas fueron Google (94,6%) y YouTube (41,7%). La mayoría indicó facilidad para usar palabras correctas (68,6%) y encontrar información (70,2%), pero dificultad para evaluar la confiabilidad (44,8%) y aplicar la información a decisiones de salud (25,9%). En el análisis ajustado, la mayor escolaridad, ser joven y tener un nivel más alto de alfabetización en salud se asociaron a la búsqueda de información de salud en línea. El uso de internet fue ampliamente reportado, pese a las dificultades para evaluar la confiabilidad y aplicar la información, destacando la necesidad de contenido de salud en línea de calidad y accesible.

## Introdução

Nas últimas décadas, a internet tornou-se uma das principais fontes de informação, incluindo informações em saúde. Estudos realizados na França ^1^ e na Indonésia ^2^ evidenciam que esse comportamento vem ocorrendo entre diferentes subgrupos populacionais, como mulheres com maior escolaridade e adolescentes. Com o aumento do uso de tecnologias digitais, ampliou-se o acesso aos conteúdos sobre doenças, tratamentos, medicamentos, diagnósticos, além de estilo de vida saudável e bem-estar ^3^. 

Os usuários são motivados pela praticidade e a gama de informações disponíveis ^4^, e por fatores como barreiras no acesso aos serviços de saúde e a percepção de conhecimento insuficiente sobre a própria doença ou tratamento ^5^ ^,^ ^6^. Uma meta-análise apontou que indivíduos com maior necessidade de informação, menor acesso a profissionais de saúde e sentimento de insegurança sobre o tratamento são mais propensos a buscar informações online ^5^. Além disso, estudo com dados de 83 países revelou que o uso da internet pode reduzir desigualdades no acesso à informação, beneficiando principalmente indivíduos com dificuldade de acesso aos serviços de saúde ^6^.

Destaca-se que nem sempre as informações são confiáveis e de fácil compreensão. Muitas vezes, carecem de embasamento científico, são produzidas sem critérios de qualidade ou apresentam viés comercial, o que dificulta a avaliação crítica e a confiabilidade. Somada a esse cenário, há a falta de habilidade em realizar pesquisas e o fenômeno da infodemia, caracterizado pela circulação massiva de informações em um período curto de tempo ^7^. O aumento do autodiagnóstico, também evidenciado entre jovens na Índia ^8^, além do acesso a tratamentos incorretos, e diminuição da cobertura vacinal, potencializada pelos movimentos antivacina em redes sociais durante a pandemia ^9^, são alguns exemplos de consequências desfavoráveis da busca de informações na internet.

Por outro lado, as informações disponíveis online podem fornecer aos indivíduos uma maior autonomia na tomada de decisões, autocuidado e conhecimento em relação à sua condição de saúde e tratamento, sendo especialmente relevante para aqueles com acesso limitado aos serviços de saúde, pois os indivíduos podem recorrer a informações confiáveis fornecidas por instituições reconhecidas. Dessa forma, o uso adequado da informação beneficia tanto o indivíduo quanto a comunidade, colaborando para a redução de custos em saúde, melhoria da qualidade de vida e de resultados em saúde ^10^ ^,^ ^11^. 

Por isso, avaliar o comportamento de uso da internet para obter informações em saúde é fundamental para a compreensão dos desafios e oportunidades que essa prática apresenta. O presente estudo tem como objetivo identificar o perfil de uso da internet e o grau de dificuldade na busca por informações em saúde por adultos, descrevendo os principais conteúdos buscados e meios utilizados, além de avaliar a associação com características sociodemográficas, médicas e com o grau de letramento em saúde.

## Metodologia

### Delineamento do estudo

Este estudo é um inquérito domiciliar multicêntrico, derivado de uma pesquisa fonte voltada à avaliação da prevalência e dos fatores associados ao letramento em saúde inadequado. Este trabalho utiliza dados da pesquisa fonte para investigar o uso da internet na busca por informações em saúde. Domicílios particulares permanentes localizados nas áreas urbanas do Sul do Brasil, que incluem Porto Alegre (capital do Rio Grande do Sul), Santa Maria e Pelotas, bem como do Centro-oeste, que incluem Campo Grande (capital do Mato Grosso do Sul), e Dourados, foram selecionados aleatoriamente. Os municípios, por sua vez, foram escolhidos por conveniência, a partir da formação de um grupo de pesquisadores de universidades públicas com experiência e/ou interesse no tema. Assim, foram escolhidos os municípios sede da Universidade Federal do Rio Grande do Sul (UFRGS), Universidade Federal de Pelotas (UFPel) e Universidade Federal de Santa Maria (UFSM) e dois *campi* da Universidade Estadual de Mato Grosso do Sul (UEMS), Campo Grande e Dourados.

### Critérios de inclusão e exclusão

Foram incluídos indivíduos com idade igual ou superior a 18 anos, que sabiam ler e escrever, e residentes em domicílios nos municípios citados. Os indivíduos que possuíam alguma limitação visual ou auditiva, que não eram fluentes na língua portuguesa ou que possuíam outra condição que os impedisse de responder a entrevista foram excluídos do estudo.

### Tamanho da amostra

Para a pesquisa fonte, foi estimada uma amostra de 1.600 entrevistas distribuídas entre os cinco municípios, considerando a proporção esperada de baixo letramento em saúde (50%). Calculou-se também o tamanho amostral necessário para analisar o desfecho de interesse do presente estudo. Nesse caso, foram estimadas 246 entrevistas, com base na proporção esperada do desfecho uso da internet para buscar informações em saúde (80%) ^12^, nível de 95% de confiança e precisão de 10%. Os cálculos foram realizados por meio do software Epi Info (https://www.cdc.gov/epiinfo/index.html), utilizando a função *Statcalc*.

### Processo de amostragem

O sorteio da amostra ocorreu por conglomerado em três estágios (setor censitário, quadra e domicílio), sendo os dois primeiros com probabilidade proporcional ao tamanho do conglomerado e o terceiro por amostra aleatória simples. Em cada município, foram sorteados 30 setores, duas quadras em cada setor, assim como 13 domicílios por quadra para as capitais e nove para as demais cidades. O sorteio das quadras foi realizado para melhorar a logística de campo e otimizar os recursos disponíveis.

Apenas uma pessoa por domicílio foi entrevistada e, quando mais de um morador estava disponível, optou-se por priorizar homens mais jovens e de maior escolaridade, devido à dificuldade de encontrá-los em seus domicílios.

Os entrevistadores receberam treinamento específico, seguiram procedimentos de abordagem padronizados e repetiram as visitas em diferentes horários e dias da semana, na tentativa de aumentar as taxas de resposta. Para minimizar o viés de seleção da pesquisa, foram calculados pesos de pós-estratificação antes da análise dos dados, utilizando o método rake. Esse procedimento minimiza os vieses de resposta e de seleção, devido à baixa taxa de resposta, utilizando as variáveis sexo, idade e escolaridade disponíveis na amostra e na população de referência para o cálculo dos pesos ^13^. Dessa maneira, esse peso iguala a distribuição da amostra ao da população. Os dados da população na capital e no interior do Rio Grande do Sul e do Mato Grosso do Sul foram extraídos da *Pesquisa Nacional por Amostra de Domicílios Contínua* do quarto trimestre de 2023 ^14^. Esses pesos foram calculados no programa Stata (https://www.stata.com), usando o pacote SURVWGT, sendo o peso de delineamento da amostra utilizado para execução do pacote ^15^.

### Pré-testes, estudo piloto e treinamento de entrevistadores

As perguntas do questionário foram formuladas a partir de revisão da literatura e testadas pela equipe de pesquisa para avaliar a consistência, clareza e relevância das perguntas, além da realização de estudo piloto em Porto Alegre para testar os procedimentos de amostragem, técnicas de abordagem aos moradores, adequabilidade do questionário e identificar inconsistências. Foi realizado o treinamento dos entrevistadores dos municípios participantes, com a disponibilização de manuais de campo e materiais de apoio. A divulgação do projeto para a população ocorreu com a entrega de folhetos informativos e canetas, notícias divulgadas em rádio e jornal, e em algumas unidades básicas de saúde (UBS) dos locais sorteados.

### Instrumento de coleta de dados 

O instrumento de coleta de dados foi disponibilizado nos *smartphones* dos entrevistadores pelo aplicativo REDCap (https://redcapbrasil.com.br/). A coleta de dados ocorreu de outubro de 2023 a março de 2024 nos cinco municípios. O questionário foi aplicado por meio de entrevista estruturada, em que o entrevistador lia as perguntas e registrava as respostas no aplicativo.

Os desfechos principais foram a busca por informações em saúde online e a autopercepção de obstáculos relacionados a esse comportamento, avaliado por meio de escala Likert de 5 pontos, que considerou as etapas de busca, obtenção, confiabilidade e uso da informação. Além disso, também foram investigados aspectos do uso da internet, como acesso, tempo diário de uso, meios utilizados (WhatsApp, Instagram, Twitter, Facebook, TikTok, Google, blogs, YouTube e aplicativos da área da saúde) e tipos de informação buscada, sendo relacionadas aos tópicos de prevenção, diagnóstico, tratamento e prognóstico.

As variáveis independentes abrangem questões relacionadas ao sexo (feminino, masculino); idade em anos completos; escolaridade, a partir do grau de estudo concluído (0-8 anos, 9-11 anos, 12 anos ou mais); autodeclaração de raça (branca, preta, amarela, parda, indígena) ^16^; classificação econômica (A, B, C, D, E) ^17^; viver com companheiro(a) (sim, não); pessoa de referência para dúvidas relacionadas à saúde no domicílio (próprio entrevistado, companheiro(a), mãe, filhos(as), pai, outros); autorrelato de doenças crônicas (diabetes, hipertensão, outra doença crônica com duração superior a 6 meses); uso de medicamentos contínuos (sim, não); adesão de plano de saúde particular (sim, não); autopercepção de saúde (muito boa, boa, regular, ruim, muito ruim); e trabalhar ou estudar na área da saúde (sim, não).

O letramento em saúde, medido por meio do *European Health Literacy Survey Questionnaire Short Form* (HLS-EU-Q6), validado para o português brasileiro, avalia as competências individuais na compreensão, avaliação e aplicação de informações relacionadas com a saúde, integrando três domínios: cuidados de saúde, promoção da saúde e prevenção de doenças ^18^. A partir da autoavaliação do indivíduo, o nível de letramento em saúde é classificado em inadequado (pontuação ≤ 2), problemático (> 2 e < 3) e suficiente (pontuação ≥ 3) ^18^. 

A classificação econômica foi definida de acordo com o Critério de Classificação Econômica da Associação Brasileira de Empresas de Pesquisa (ABEP) ^17^, que atribui pontos com base na quantidade de eletrodomésticos, veículos e funcionários no domicílio, além do acesso a serviços públicos e escolaridade da pessoa que contribui com a maior parte da renda do domicílio. A soma da pontuação enquadra os indivíduos em seis categorias: A, B1, B2, C1, C2 e DE. Neste artigo, agrupamos as categorias em A/B, C e D/E. 

Para identificar a pessoa de referência para dúvidas relacionadas à saúde no domicílio, o entrevistado respondia à pergunta “Quem é a pessoa que todos procuram nesta casa quando o assunto é saúde?”.

O controle de qualidade dos dados incluiu supervisão dos entrevistadores, análise diária das informações coletadas e reentrevistas telefônicas de questionários com inconsistências (duplicidade, valores ausentes e respostas incoerentes), em que foram reaplicadas questões-chave selecionadas pela equipe de pesquisa.

### Análise de dados

As análises estatísticas foram realizadas no módulo *survey* disponível no software RStudio (https://rstudio.com/), versão 2024.12.0+467, utilizando as variáveis delineamento da amostra, unidade primária de amostragem e pesos de pós-estratificação para obtenção das estatísticas, que incluíram descrições por médias e desvios padrão (quando aplicável), frequências relativas ajustadas pelos pesos de pós-estratificação e seus respectivos intervalos de 95% de confiança (IC95%).

A associação entre as variáveis foi testada pelo teste χ² de Pearson (Rao-Scott) ajustado para amostras complexas. As análises de regressão de Poisson com variância robusta foram expressas por razões de prevalência (RP), com nível de 5% de significância. As variáveis (sexo, idade, raça, escolaridade, classificação econômica, moradia compartilhada com companheiro(a), autopercepção de saúde, autorrelato de doenças crônicas, uso de medicamentos contínuos e grau de letramento em saúde) foram analisadas individualmente, e aquelas com p < 0,20 foram incluídas no modelo inicial. Em seguida, utilizou-se o método *backward*, excluindo-se variáveis com p > 0,05, conforme o teste de Wald.

As respostas dos questionários com dados ausentes não foram analisadas. A escala Likert foi agrupada em fácil (fácil e muito fácil), nem fácil nem difícil e difícil (difícil e muito difícil).

### Aspectos éticos

O Termo de Consentimento Livre Esclarecido (TCLE) foi assinado por todos os participantes que concordaram em participar da entrevista. A pesquisa foi aprovada pelos Comitês de Ética em Pesquisa da Universidade Federal do Rio Grande do Sul (UFRGS, nº 52872721.0.0000.5347), e das universidades participantes da pesquisa (Universidade Federal de Santa Maria, nº 52872721.0.3001.5346; Universidade Federal de Pelotas, nº 52872721.0.3002.5317 e Universidade Estadual de Mato Grosso do Sul, nº 52872721.0.3003.8030).

## Resultados

**Figura 1:**
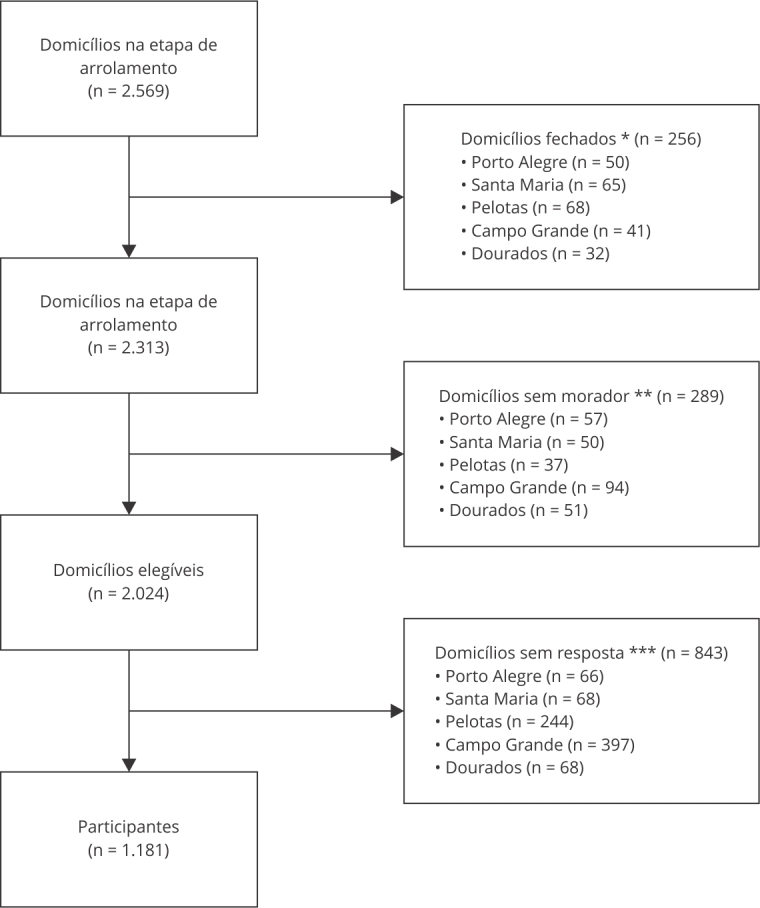
Fluxograma dos participantes incluídos no estudo sobre letramento em saúde residentes em municípios das regiões Sul e Centro-oeste, Brasil, 2024.

No total, 1.181 pessoas participaram da pesquisa, responderam às perguntas sobre acesso à internet e foram incluídas nas análises (Figura 1). Os principais motivos para os domicílios sem resposta incluíram a falta de tempo por parte dos moradores, insegurança quanto ao entrevistador, receio de compartilhar informações ou em assinar o TCLE, falta de interesse pela pesquisa, problemas de saúde na família, dificuldades de locomoção e até obstáculos relacionados à leitura. Entre os 1.181 participantes, 92,3% tinham acesso à internet, dos quais 77,1% utilizavam a internet para buscar informações sobre saúde.

* Morador não estava presente no momento da entrevista; ** Domicílio em reforma, aluguel ou com placa de venda; *** Falta de tempo, insegurança em relação ao entrevistador, receio em compartilhar informações ou assinar o Termo de Consentimento Livre e Esclarecido (TCLE), desinteresse, problemas de saúde na família, dificuldades de locomoção e limitações de leitura.

A maioria dos participantes era do sexo feminino, com idade entre 18 e 39 anos, predominantemente branca, com 12 anos de estudo ou mais, pertencentes as classes A/B, com plano de saúde, com companheiro, sendo o próprio entrevistado a pessoa de referência para consultas de saúde, com autopercepção de saúde boa ou muito boa, e apresentando letramento em saúde problemático. Menos da metade dos participantes trabalhava ou estudava na área da saúde, possuía diagnóstico de doença crônica e usava medicamentos de forma contínua. Esse perfil também foi observado entre os indivíduos que buscaram informações sobre saúde online, com diferença estatisticamente significativa para idade, escolaridade, nível econômico, plano de saúde, trabalhar ou estudar na área da saúde, pessoa de referência para consultas de saúde e grau de letramento em saúde (p < 0,05) (Tabela 1).

**Tabela 1:** Dados sociodemográficos e relacionados à saúde dos participantes. Regiões Sul e Centro-oeste, Brasil, 2024.

Características	Total		Usuários da internet		Valor de p *	Busca de informações em saúde online		Valor de p *
	n ** (% ***)	IC95% ***	n ** (% ***)	IC95% ***		n ** (% ***)	IC95% ***	
Respondentes	1.181 (100,0)	-	1.066 (92,3)	90,3-94,4		818 (77,1)	72,4-81,8	
Sexo					0,143			0,146
Feminino	709 (50,8)	46,8-54,8	648 (51,4)	47,1-55,7		517 (53,4)	48,5-58,2	
Masculino	472 (49,2)	45,2-53,2	418 (48,6)	44,3-52,8		301 (46,6)	41,7-51,5	
Idade [média (DP)]	44,9 (± 16,9)		43,1 (± 16,0)			41,2 (± 14,9)		
Faixa etária (anos)					< 0,001			< 0,001
18-39	409 (43,8)	39,0-48,5	404 (47,0)	42,0-52,1		340 (51,2)	45,4-57,0	
40-59	425 (31,9)	28,3-35,5	404 (33,2)	29,4-37,0		315 (32,5)	27,8-37,2	
≥ 60	347 (24,3)	20,5-28,0	258 (19,7)	16,0-23,5		163 (16,2)	12,3-20,2	
Raça e etnia				0,240			0,148	
Branca	742 (66,3)	61,8-70,8	678 (67,1)	62,4-71,9		535 (68,9)	63,8-74,0	
Parda	285 (21,1)	17,5-24,8	252 (20,6)	16,7-24,4		178 (18,9)	14,9-22,8	
Preta	133 (9,4)	7,0-11,8	118 (9,0)	6,7-11,4		88 (8,0)	5,3-10,7	
Amarela	11 (1,6)	0,0-3,2	9 (1,6)	0,0-3,4		9 (2,1)	0,0-4,3	
Indígena	3 (0,8)	0,0-2,0	3 (0,8)	0,0-2,1		3 (1,1)	0,0-2,8	
Escolaridade, média (DP)	11,6 (± 4,6)		12,1 (± 4,2)			12,8 (± 3,7)		
Escolaridade (anos completos de estudo)					< 0,001			< 0,001
0-8	307 (26,5)	21,8-31,3	224 (21,9)	17,4-26,5		123 (16,1)	11,5-20,8	
9-11	525 (14,0)	11,9-16,0	501 (14,6)	12,3-16,8		411 (15,5)	12,9-18,1	
12 ou mais	349 (59,5)	54,2-64,8	341 (63,5)	58,2-68,7		284 (68,4)	63,0-73,7	
Classificação econômica					<0,001			0,021
A/B	597 (60,1)	54,9-65,3	573 (63,2)	58,1-68,4		474 (66,0)	60,4-71,6	
C	483 (32,2)	27,6-36,7	422 (30,7)	26,1-35,4		303 (29,1)	24,0-34,2	
D/E	101 (7,7)	5,3-10,2	71 (6,0)	3,8-8,3		41 (4,9)	2,6-7,2	
Possui plano de saúde	589 (56,9)	51,4-62,3	546 (58,6)	53,3-64,0	0,003	447 (63,2)	58,1-68,3	0,003
Vive com companheiro(a)	875 (75,7)	71,1-80,3	786 (75,3)	70,4-80,2	0,302	599 (75,5)	69,7-81,4	0,804
Trabalha ou estuda na área da saúde	153 (14,2)	10,9-17,5	148 (15,1)	11,6-18,6	< 0,001	130 (17,1)	13,0-21,3	0,019
Pessoa de referência para dúvidas sobre saúde					< 0,001			0,009
O próprio entrevistado	585 (59,3)	53,7-64,9	549 (59,8)	53,8-65,8		434 (59,5)	52,7-66,2	
Companheiro(a)	154 (16,2)	12,4-20,0	143 (16,3)	12,3-20,2		103 (15,0)	10,5-19,4	
Mãe	87 (10,0)	5,8-14,2	86 (10,5)	6,1-14,9		71 (11,6)	6,3-16,8	
Filhos(as)	43 (4,0)	2,7-5,4	28 (3,5)	1,8-4,3		14 (1,9)	0,8-3,1	
Pai	22 (3,2)	1,0-5,4	22 (3,4)	1,1-5,7		20 (4,2)	1,3-7,2	
Outros	66 (7,2)	4,4-10,1	57 (7,0)	4,1-9,9		45 (7,8)	4,4-11,2	
Autopercepção de saúde					0,007			0,110
Muito boa/Boa	831 (72,7)	68,9-76,5	762 (73,8)	69,9-77,6		594 (76,2)	71,6-80,8	
Regular	311 (25,3)	21,5-29,0	275 (24,8)	20,9-28,6		203 (22,5)	17,8-27,2	
Muito ruim/Ruim	36 (2,0)	1,1-2,9	26 (1,5)	0,7-2,2		18 (1,33)	0,5-2,2	
Diagnóstico de doença crônica ^#^	335 (26,8)	22,7-31,0	297 (26,3)	22,1-30,5	0,373	231 (26,3)	21,5-31,0	0,495
Uso de medicamentos contínuos ^##^	590 (44,9)	40,2-49,5	516 (43,5)	38,8-48,3	0,006	382 (42,7)	37,5-47,9	0,354
Grau de letramento em saúde					0,002			< 0,001
Suficiente	258 (26,7)	22,1-31,2	244 (27,7)	22,9-32,5		209 (30,9)	25,5-36,3	
Problemático	742 (61,7)	55,7-67,7	687 (62,6)	56,5-68,7		528 (62,5)	56,8-68,3	
Inadequado	160 (11,5)	8,2-14,9	123 (9,7)	6,4-12,9		75 (6,6)	4,0-9,1	

DP: desvio padrão; IC95%: intervalo de 95% de confiança.

* Teste χ^2^ de Pearson com correção de Rao-Scott;

** Não ponderado pelos pesos amostrais;

*** N = 5.000.000. Percentuais ponderados pelos pesos amostrais;

^#^ Participante referiu ter diagnóstico de ao menos uma doença crônica;

^##^ Participante referiu usar ao menos um medicamento para tratamento de doença crônica.

Na análise bruta, a busca por informações sobre saúde online associou-se positivamente com sexo feminino, idade abaixo de 60 anos, raça branca, escolaridade superior a 8 anos, classificação econômica A, B ou C, viver com companheiro e grau de letramento em saúde suficiente ou problemático; e negativamente com autopercepção de saúde regular, muito ruim ou ruim e uso contínuo de medicamentos. Na análise ajustada, as associações positivas verificadas na análise bruta mantiveram-se (Tabela 2), exceto a classificação econômica A, B ou C (p = 0,244).

**Tabela 2:** Razões de prevalências (RP) brutas e ajustadas dos fatores independentemente associados ao uso da internet para a busca de informação em saúde. Regiões Sul e Centro-oeste, Brasil, 2024.

Características	Usuários da internet que buscam informações em saúde					
	RP bruta *	IC95%	Valor de p	RP ajustada *	IC95%	Valor de p
Sexo [n = 1.065 **]						
Feminino	1,08	1,07-1,08	< 0,001	1,09	1,08-1,10	< 0,001
Masculino	1,00	-		1,00	-	
Faixa etária (anos) [n = 1.065 **]						
18-39	1,33	1,31-1,34	< 0,001	1,25	1,23-1,27	< 0,001
40-59	1,19	1,18-1,20		1,15	1,14-1,17	
≥ 60	1,00	-		1,00	-	
Raça [n = 1.065 **]						
Branca	1,09	1,08-1,09	< 0,001	1,04	1,03-1,05	< 0,001
Amarelo, indígena, parda e preta	1,00	-		1,00	-	
Escolaridade (anos completos de estudo) [n = 1.065 **]						
0-8	1,00	-		1,00	-	
9-11	1,45	1,42-1,47	< 0,001	1,33	1,31-1,35	< 0,001
12 ou mais	1,47	1,45-1,49		1,34	1,32-1,36	
Classificação econômica [n = 1.065 **]						
A/B	1,28	1,27-1,30	< 0,001			
C	1,16	1,15-1,17				
D/E	1,00	-				
Vive com companheiro(a) [n = 1.065 **]						
Sim	1,01	1,01-1,02	< 0,001	1,04	1,03-1,05	< 0,001
Não	1,00	-		1,00	-	
Autopercepção de saúde [n = 1.062 **]						
Muito boa/Boa	1,00	-		1,00	-	
Regular	0,89	0,88-0,89	< 0,001	0,96	0,95-0,98	< 0,001
Muito ruim/Ruim	0,88	0,87-0,89		1,04	1,03-1,05	
Diagnóstico de doença crônica [n = 1.060 **]						
Sim	1,00	0,99-1,00	0,506			
Não	1,00	-				
Uso de medicamentos contínuos [n = 1.060 **]						
Sim	0,96	0,96 - 0,97	< 0,001	1,05	1,04-1,06	< 0,001
Não	1,00	-		1,00	-	
Grau de letramento em saúde [n = 1.053 **]						
Suficiente	1,65	1,58-1,73	< 0,001	1,40	1,34-1,47	< 0,001
Problemático	1,48	1,40-1,55		1,32	1,25-1,39	
Inadequado	1,00	-		1,00	-	

IC95%: intervalo de 95% de confiança.

* Razão de prevalência ajustada para sexo, faixa etária, raça e etnia, escolaridade, vive com companheiro(a), autoavaliação de saúde, uso de medicamentos contínuos e grau de letramento em saúde;

** Não ponderado pelos pesos amostrais.

O tempo médio diário de uso da internet relatado foi de 5 horas (5,14 ± 3,55 horas), com o menor número de indivíduos utilizando a internet por menos de 1 hora por dia (11,3%; IC95%: 5,5-17,1). As ferramentas mais utilizadas para realizar buscas sobre saúde na internet foram, nessa ordem, plataformas de busca na internet (como Google, Bing e Yahoo) (94,6%; IC95%: 91,2-98,0), YouTube (41,7%; IC95%: 36,1-47,3), aplicativos de saúde (24,3%; IC95%: 19,2-29,5), Instagram (23,5%; IC95%: 18,4-28,7), WhatsApp (23,2%; IC95%: 17,2-29,1), Facebook (16,7%; IC95%: 11,9-21,5), blogs (10,9%; IC95%: 7,0-14,8), TikTok (8,1%; IC95%: 4,6-11,5) e Twitter (3,7%; IC95%: 0,9-6,5).

**Figura 2:**
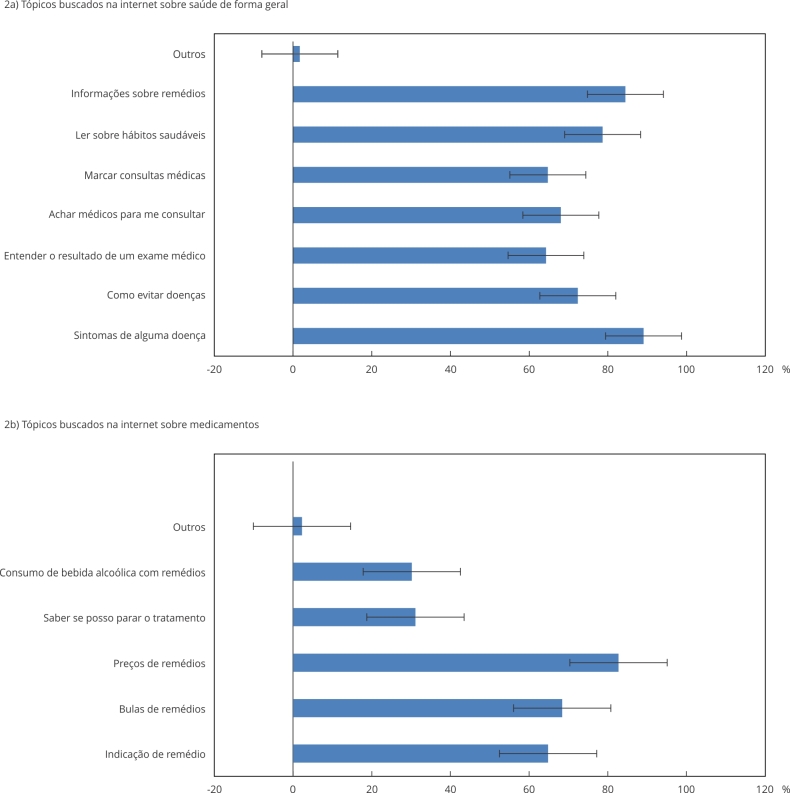
Temas relacionados à saúde pesquisados na internet pelos entrevistados.

Os três principais temas pesquisados na internet foram relacionados a sintomas (89,1%; IC95%: 86,2-92,0), uso de medicamentos (84,5%; IC95%: 79,7-89,2) e conteúdo sobre hábitos saudáveis (78,7%; IC95%: 73,4-83,9) (Figura 2a); e os temas mais pesquisados sobre medicamentos foram preços (82,8%; IC95%: 78,2-87,3), bulas (68,4%; IC95%: 62,7-74,2) e indicação (64,8%; IC95%: 59,4-70,2) (Figura 2b).

Quando perguntados sobre o nível de dificuldade para usar as palavras certas para encontrar as informações que procuravam na internet, a maioria dos entrevistados considerou essa tarefa fácil ou muito fácil (68,6%; IC95% :63,6-73,7). A maioria também relatou facilidade (fácil ou muito fácil) em encontrar as informações que procuravam (70,2%; IC95%: 64,9-75,4). No entanto, 44,8% (IC95% 38,8-50,8) relatou que é difícil ou muito difícil decidir se a informação é confiável e 25,9% (IC95%: 20,8-31,1) relatou que é difícil ou muito difícil usar as informações encontradas para tomar decisões em saúde. A ausência de respostas foi baixa e não influenciou as estimativas, variando entre 0,3% e 0,8% (Tabela 3).

**Tabela 3:** Nível de dificuldade autorreferido para buscas na Internet sobre saúde. Regiões Sul e Centro-oeste, Brasil, 2024.

Nível de dificuldade	Fácil/Muito fácil	Nem fácil nem difícil	Difícil/Muito difícil
	% * (IC95%)	% * (IC95%)	% * (IC95%)
Qual o seu nível de dificuldade para...			
Usar as palavras certas para encontrar as informações que procura? [n = 815]	68,9 (63,8-74,0)	22,4 (17,9-26,8)	8,7 (6,1-11,2)
Achar a informação que você está procurando? [n = 815]	70,5 (65,1-75,8)	19,6 (15,0-24,1)	9,9 (6,7-13,2)
Decidir se a informação é confiável? [n = 815]	29,1 (23,8-34,3)	25,9 (20,6-31,3)	45,0 (39,0-51,0)
Usar as informações que você achou para tomar decisões sobre sua saúde? [n = 815]	51,9 (46,2-57,6)	21,9 (18,1-25,8)	26,2 (21,0-31,3)

IC95%: intervalo de 95% de confiança.

* Percentuais ponderados pelos pesos amostrais.

A relação entre o nível de dificuldade na busca por informações sobre saúde online e o grau de letramento em saúde é apresentada na Tabela 4. Os indivíduos com letramento em saúde problemático e inadequado apresentaram maior dificuldade em pesquisar e encontrar as informações procuradas, bem como decidir sobre a confiabilidade das informações e usá-las para tomar decisões em saúde, em comparação com aqueles com letramento em saúde suficiente (Tabela 4).

**Tabela 4:** Nível de letramento em saúde, avaliado pelo *European Health Literacy Survey Questionnaire Short Form* (HLS-EU-Q6) em relação ao nível de dificuldade para buscar informações em saúde. Regiões Sul e Centro-oeste, Brasil, 2024.

Nível de dificuldade	Fácil/Muito fácil	Nem fácil nem difícil	Difícil/Muito difícil	Valor de p *
	% ** (IC95%)	% ** (IC95%)	% ** (IC95%)	
Usar as palavras certas para encontrar as informações que procura [n = 809 ***]				< 0,001
Suficiente	79,8 (74,5-85,2)	17,1 (8,3-25,9)	3,1 (0,0-18,5)	
Problemático	67,2 (61,2-73,3)	23,7 (14,5-32,9)	9,1 (0,0-23,9)	
Inadequado	35,8 (32,6-39,0)	32,8 (28,7-37,0)	31,4 (19,9-43,0)	
Achar a informação que está procurando [n = 810 ***]				< 0,001
Suficiente	83,4 (78,1-88,6)	15,4 (7,2-23,6)	1,2 (0,0-17,2)	
Problemático	66,9 (59,8-74,0)	20,2 (12,0-28,4)	12,9 (0,0-26,4)	
Inadequado	43,7 (38,8-48,7)	32,7 (31,4-33,9)	23,6 (11,0-36,2)	
Decidir se a informação é confiável [n = 807 ***]				< 0,001
Suficiente	53,3 (45,9-60,7)	26,5 (18,0-34,9)	20,2 (5,9-34,5)	
Problemático	19,4 (14,2-24,5)	26,0 (15,7-36,4)	54,6 (43,2-66,0)	
Inadequado	8,5 (0,7-16,1)	22,7 (14,3-31,2)	68,8 (53,3-84,4)	
Usar as informações para tomar decisões sobre saúde [n = 801 ***]				< 0,001
Suficiente	78,5 (72,9-84,0)	12,5 (6,8-18,2)	9,0 (0,0-23,5)	
Problemático	42,6 (34,5-50,6)	25,8 (17,3-34,3)	31,6 (18,1-45,1)	
Inadequado	18,0 (11,0-25,0)	26,9 (20,4-33,4)	55,1 (39,7-70,4)	

IC95%: intervalo de 95% de confiança.

* Teste χ^2^ de Pearson com correção de Rao-Scott;

** Percentuais ponderados pelos pesos amostrais;

*** Não ponderado pelos pesos amostrais.

## Discussão

A maioria dos participantes relatou buscar informações em saúde na internet, com cerca de 8 em cada 10 usuários da internet realizando buscas sobre saúde (77,1%). Outros estudos envolvendo a internet como fonte de informação em saúde mostram dados semelhantes, com taxas acima de 65%, principalmente para países desenvolvidos ^1^ ^,^ ^2^ ^,^ ^19^ ^,^ ^20^. O resultado encontrado é elevado em comparação com outros estudos brasileiros, que demonstram proporções em torno de 65% ^21^ ^,^ ^22^. No entanto, esses estudos não são muito recentes, com dados coletados em 2014 e 2018, respectivamente, o que pode não refletir mudanças no acesso e nos padrões da internet ao longo do tempo.

De forma geral, o comportamento de busca por informações em saúde online foi mais frequente entre as mulheres, indivíduos mais jovens, de cor branca, vivendo com companheiro, com pior autopercepção de saúde, em uso de medicamentos contínuos, com maior nível educacional e elevado grau de letramento em saúde, em conformidade com estudos anteriores ^1^ ^,^ ^21^ ^,^ ^23^.

Indivíduos com nível educacional e grau de letramento em saúde elevados referiram uso da internet com mais frequência em comparação com indivíduos menos escolarizados e com letramento em saúde mais baixo. Essa diferença pode ser explicada devido a essas características favorecerem a percepção de risco da própria doença e a noção de insuficiência de conhecimento sobre seu problema de saúde ^6^. Além disso, alguns estudos apontam que indivíduos com níveis reduzidos de letramento em saúde apresentam dificuldades para navegar em sites, compreender termos médicos e avaliar a credibilidade de fontes de informação ^24^ ^,^ ^25^, o que dificulta a operação de busca por informações de forma digital por essa população.

De forma similar, os indivíduos mais jovens usam mais a internet para buscar informações em saúde, provavelmente devido à familiaridade e facilidade de uso de tecnologias digitais ^26^. Em contraste, os idosos enfrentam maior exclusão digital ^27^ devido a barreiras físicas do próprio processo de envelhecimento, como visão e habilidades motoras prejudicadas; falta de conhecimento em utilizar tecnologias e entender termos digitais; percepções negativas e estigma sobre a internet, e medo e desconfiança de informações obtidas online ^28^. 

As variáveis relacionadas ao sexo feminino e a viver com companheiro também foram associadas ao desfecho. Em nosso estudo, a pessoa de referência para dúvidas sobre saúde foi, nessa ordem, o próprio entrevistado, o(a) companheiro(a) e a mãe. Esse fenômeno pode ser explicado por questões culturais, onde a mulher frequentemente desempenha o papel de principal cuidadora da família, assumindo maior responsabilidade por dúvidas e cuidados em saúde ^29^. Além disso, viver com um(a) companheiro(a) pode facilitar as discussões e a tomada de decisões conjuntas sobre os dados encontrados online. 

Observou-se também uma maior prevalência na busca por informações em saúde online entre os indivíduos que autoavaliaram sua saúde como ruim ou muito ruim e aqueles em uso de medicamentos contínuos. A necessidade de reconhecer efeitos adversos e obter indicações de medicamentos, assim como autogerir o seu problema de saúde de forma independente e avaliar a necessidade de consultar um especialista ^30^ podem ter influenciado esses indivíduos a buscarem na internet. 

Em nosso estudo, não verificamos maior uso da internet entre as pessoas portadoras de doenças crônicas, possivelmente porque a busca por informações pode ser realizada tanto por indivíduos com problemas de saúde crônico quanto por aqueles interessados em temas de prevenção e promoção da saúde, e ainda para outras questões, como sintomas e condições de saúde agudas ^31^. 

Da mesma forma, era esperado que indivíduos com melhores condições econômicas buscassem mais informações sobre saúde na internet ^1^ ^,^ ^32^. No entanto, o resultado encontrado pode refletir a crescente democratização do uso da internet. Em um estudo realizado na Malásia ^33^, pesquisadores observaram que o comportamento de busca por informações de saúde online era generalizado entre os pacientes, independentemente da classe socioeconômica, possivelmente devido à expansão do acesso à internet, o que também ocorre no Brasil ^34^. 

Em relação à dificuldade de busca por informações sobre saúde na internet, os entrevistados consideraram a parte operacional fácil, ou seja, usar as palavras certas e encontrar as informações que buscavam. No entanto, relataram dificuldade em decidir se as informações eram confiáveis e em aplicá-las para tomar decisões sobre sua saúde. Dúvidas sobre a credibilidade dificultam a aplicação das informações encontradas, o que pode estar relacionado ao grau de letramento em saúde. Indivíduos com maiores escores de letramento em saúde conseguem entender e usar as informações com maior facilidade, além de reconhecer informações de baixa qualidade ^35^ ^,^ ^36^. 

Em nosso estudo, os indivíduos com letramento em saúde problemático e inadequado apresentaram os maiores índices de dificuldade para buscar informações em saúde na internet, desde o uso das palavras certas até a aplicação das informações para tomar decisões. Esses resultados mostram que, quanto mais baixo o letramento em saúde do indivíduo, maiores as barreiras enfrentadas para acessar e utilizar informações em saúde online de forma independente e crítica. A relação entre o letramento em saúde e a busca por informações online é estratégica para a saúde pública, uma vez que níveis mais elevados de letramento em saúde favorecem a interpretação de conteúdos complexos, a distinção entre dados científicos e informações não verificadas, além de fortalecer a autonomia do indivíduo. Em contrapartida, níveis baixos de letramento em saúde aumentam a vulnerabilidade da população à infodemia e dificultam a tomada de decisões baseadas em evidências.

Os indivíduos com letramento em saúde adequado passam mais tempo analisando resultados de pesquisa online e conteúdos textuais, em comparação com aqueles com menores níveis de letramento em saúde ^37^, o que pode favorecer a diferenciação entre dados científicos e *fake news*. Outros aspectos que influenciam a confiança incluem sites com designs pouco amigáveis e não intuitivos ^38^, além da variação na qualidade de fontes online. Esses aspectos possuem grande relevância, pois demonstram que o nível de letramento em saúde influencia não apenas a busca por informações online, mas também a habilidade de julgar a confiabilidade e traduzi-las em práticas de autocuidado. 

Apesar das barreiras em decidir sobre a confiabilidade e aplicabilidade das informações, a internet foi citada como uma fonte de informação em saúde por 77,1% dos entrevistados. Isso pode ser explicado pela praticidade e facilidade de uso, o imediatismo e o anonimato ^39^. Um estudo realizado na China revelou que a escolha de sites sobre saúde é mais pautada pela conveniência do que pela precisão das informações ^4^. A confiança nas informações online também é modificada por diferenças culturais e geográficas. Por exemplo, um estudo demonstrou diferenças entre países, sendo os participantes chineses mais propensos a confiar e usar informações de saúde online, enquanto os norte-americanos disseram preferir obter informações por profissionais da saúde ^40^. 

Há várias formas de se obter informações em saúde na internet. Em nosso estudo, as ferramentas mais citadas foram os buscadores eletrônicos (Google, Bing, Yahoo, entre outros), seguido pelo YouTube e aplicativos de saúde. Cada ferramenta oferece padrões diferentes para obter informações: o usuário pode pesquisar ativamente por informações específicas em aplicativos e sites, ou pode recebê-las passivamente. 

Whatsapp, Instagram e Facebook, por exemplo, podem ser classificados como meios de informação passivos, já que, tradicionalmente, o usuário não realiza buscas na plataforma, mas sim recebe as informações no seu *feed* de notícias, ou por meio de envio de mensagens sobre saúde. Por outro lado, os mecanismos de pesquisa, como Google, Bing e Yahoo, além do YouTube, permitem ao usuário realizar pesquisas sobre determinado assunto. 

Isso pode ser observado em estudo realizado por Lim et al. ^41^ com adultos australianos para entender o uso das redes sociais na busca por informações em saúde. De acordo com os autores, o Google servia como ponto de partida para a busca e, em muitos casos, era seguido pelo YouTube, que auxiliava na decisão sobre a credibilidade das informações, com base no julgamento sobre o apresentador e na aparência do vídeo. As demais redes sociais, como Facebook e Instagram, eram utilizadas principalmente para receber recomendações sobre estilo de vida saudável e bem-estar. Nesse último caso, o indivíduo acompanhava o conteúdo de celebridades e influenciadores, sem procurar informações efetivamente ^41^. 

Em relação aos temas mais buscados pelos entrevistados em nosso estudo, os hábitos saudáveis, como condicionamento físico e dieta, representaram cerca de 80% das buscas ^3^ ^,^ ^4^. Isso ocorre principalmente pela preocupação crescente em se prevenir de doenças não transmissíveis, como a obesidade, hipertensão e diabetes; e o apoio social online, a partir da discussão e troca de experiências entre os internautas em fóruns e grupos ^30^. 

As informações sobre medicamentos e sintomas de doenças foram as mais procuradas. O consumo dessas informações na internet apresenta vantagens, como o gerenciamento inicial de problemas de saúde pelos indivíduos e a autossuficiência ^39^. O usuário torna-se mais ativo em relação ao seu tratamento ao buscar informações por conta própria e, consequentemente, acaba levando a uma mudança no modelo tradicional centrado no médico. Nesse modelo colaborativo, o profissional de saúde discute as informações encontradas online com o paciente e o ajuda a aplicá-las. Um estudo demonstrou que indivíduos que buscam informações de saúde online e as discutem com um médico são 2,54 vezes mais propensos a modificar seu estilo de vida ^42^, tornando a internet uma ferramenta complementar e não uma substituta aos profissionais da saúde. 

Contudo, a busca por sintomas e indicação de medicamentos na internet também levantam preocupações, como o autodiagnóstico e a decisão de iniciar um tratamento por conta própria. Em pesquisa realizada nos Estados Unidos, cerca de um terço dos entrevistados relatou se autodiagnosticar utilizando informações online ^43^. Esse cenário é especialmente frequente entre jovens com baixa escolaridade ^44^ e em situações em que o acesso aos serviços de saúde é restrito ou necessariamente pago ^6^, a exemplo da Hungria, Polônia e Estados Unidos, que não possuem um sistema de saúde universal. 

Nesses casos, o usuário acaba utilizando a internet como alternativa para solucionar os seus problemas de saúde, o que também ocorre, de forma similar, com a prática da automedicação. Em um estudo realizado a partir de questionário online, mais da metade dos entrevistados (59,8%) disseram obter informações em saúde na internet para se automedicar e, dentre eles, cerca de metade (54,5%) fizeram uso de medicamentos sem consultar um médico e 11,6% se automedicaram para condições graves, como diabetes e câncer ^45^. Isso ocorre por fatores como o excesso de confiança e a prática de autocuidado ^46^. A quantidade de informações pesquisadas online também parece influenciar as decisões sobre saúde, possivelmente devido à percepção do indivíduo de que assuntos repetidos na internet são necessariamente verdadeiros. 

Além disso, vale ressaltar que cerca de um terço dos entrevistados relatou realizar buscas na internet para saber se poderia interromper o tratamento. Esse comportamento pode refletir preocupações com os efeitos adversos dos medicamentos ^47^ ou ainda indicar uma possível motivação relacionada à economia financeira na compra de medicamentos ^48^. Esses fatores podem estar relacionados a mudanças no esquema terapêutico ou até mesmo à desistência do tratamento. 

Entre as limitações deste estudo, destacamos a não aleatoriedade no último estágio de seleção dentro do domicílio, diminuindo a representatividade da população. O sorteio das quadras no segundo estágio, realizado para melhorar a logística de campo e otimizar os recursos disponíveis, também pode ser considerado uma limitação, devido ao potencial efeito de conglomeração decorrente da alta homogeneidade dos domicílios dentro das quadras. Ainda que esta limitação não possa ser completamente corrigida, utilizamos pesos de pós-estratificação para minimizar os vieses de seleção e de resposta, devido à baixa taxa de resposta. 

Por fim, é importante salientar que os resultados deste estudo irão contribuir para um melhor entendimento sobre as necessidades, interesses e preocupações relacionadas à saúde pelos usuários. Além disso, estratégias futuras devem ser pensadas para promover a certificação de sites seguros e confiáveis, e com linguagem acessível para a população. Estudos que avaliem o uso de informações em saúde online podem auxiliar profissionais de saúde e órgãos públicos sobre a importância da educação em saúde, especialmente relacionada às informações disponíveis na internet. 

Ademais, os achados deste estudo, conduzido em período anterior à popularização da inteligência artificial, oferecem um ponto de referência para comparações em estudos futuros sobre o acesso e uso de informações em saúde na internet, considerando que a introdução de novas tecnologias pode modificar as dificuldades enfrentadas pelos indivíduos na busca por informações. Os resultados do estudo reforçam a importância de discutir o letramento digital em saúde como recurso essencial de autocuidado e criticidade dos usuários com as informações disponível na internet, especialmente diante do desafio da infodemia.

## Conclusão

A maioria dos entrevistados relatou utilizar a internet como fonte de informação sobre saúde, utilizando principalmente mecanismos de busca para obter dados sobre sintomas e uso de medicamentos. Esse comportamento foi observado principalmente entre indivíduos mais jovens, e com mais de 8 anos de escolaridade, apesar da dificuldade em decidir se as informações são confiáveis e aplicá-las em suas vidas diárias. Os resultados do estudo mostram que a internet pode auxiliar os usuários em suas decisões de saúde e destacam a necessidade de informações online confiáveis e de qualidade para os usuários.

## Data Availability

Os dados de pesquisa estão disponíveis mediante solicitação à autora de correspondência
